# 1-Methyl-4*H*-3,1-benzoxazine-2,4(1*H*)dione

**DOI:** 10.1107/S1600536810006094

**Published:** 2010-02-20

**Authors:** Nicholas P. Deifel, Emily Cherney, David A. Hunt, Benny C. Chan

**Affiliations:** a Department of Chemistry, The George Washington University, 725 21st Street, NW, Washington, DC 20052, USA; b Department of Chemistry, The College of New Jersey, 2000 Pennington Rd, Ewing, NJ 08628, USA

## Abstract

In its crystal structure, the title compound, C_9_H_7_NO_3_, forms π-stacked dimers, with a centroid–centroid distance of 3.475 (5) Å between the benzenoid and the 2,4 dicarbonyl oxazine rings. These dimers then form staircase-like linear chains through further π-stacking between the benzenoid rings [centroid–centroid distance of 3.761 (2) Å]. The methyl-H atoms are disordered due to rotation about the C—N bond and were modeled with equal occupancy.

## Related literature

The title compound is a key inter­mediate for the synthesis of a variety of compounds, see: Coppola (1980 ▶); Kappe & Stadlbauer (1981 ▶); Shvekhgeimer (2001 ▶). Isatoaic anhydrides are important for the synthesis of a variety of commercial compounds. The crystal structures of two other isotoic anydrides have been reported: for the brominated 6-bromo-2*H*-3,1-benzoxazine-2,4(1*H*)-dione, see: Lubini & Wouters (1996 ▶) and for the unfunctionalized 2*H*-3,1-benzoxazine-2,4(1*H*)-dione, see: Kashino *et al.* (1978 ▶).
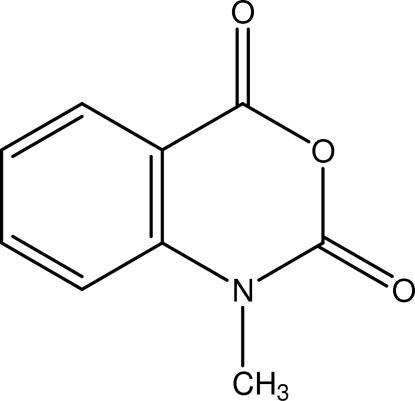


## Experimental

### 

#### Crystal data

C_9_H_7_NO_3_
*M* *_r_* = 177.16
Monoclinic, 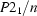

*a* = 7.632 (2) Å
*b* = 8.818 (2) Å
*c* = 11.719 (3) Å
β = 93.599 (4)°
*V* = 787.1 (4) Å^3^
*Z* = 4
Mo *K*α radiation
μ = 0.11 mm^−1^
*T* = 296 K
0.5 × 0.5 × 0.4 mm

#### Data collection

Bruker APEXII CCD diffractometer
Absorption correction: multi-scan (*SADABS*; Bruker, 2008 ▶) *T*_min_ = 0.902, *T*_max_ = 0.934
13548 measured reflections
2191 independent reflections
1532 reflections with *I* > 2σ(*I*)
*R*_int_ = 0.029

#### Refinement

*R*[*F* ^2^ > 2σ(*F* ^2^)] = 0.052
*wR*(*F* ^2^) = 0.160
*S* = 1.07
2191 reflections
118 parameters
H-atom parameters constrained
Δρ_max_ = 0.20 e Å^−3^
Δρ_min_ = −0.21 e Å^−3^

### 

Data collection: *APEX2* (Bruker, 2008 ▶); cell refinement: *SAINT-Plus* (Bruker, 2008 ▶); data reduction: *SAINT-Plus* (Bruker, 2008 ▶); program(s) used to solve structure: *SHELXS97* (Sheldrick, 2008 ▶); program(s) used to refine structure: *SHELXL97* (Sheldrick, 2008 ▶); molecular graphics: *CrystalMaker* (CrystalMaker, 2009 ▶); software used to prepare material for publication: *WinGX* (Farrugia, 1999 ▶).

## Supplementary Material

Crystal structure: contains datablocks I, global. DOI: 10.1107/S1600536810006094/fj2280sup1.cif

Structure factors: contains datablocks I. DOI: 10.1107/S1600536810006094/fj2280Isup2.hkl

Additional supplementary materials:  crystallographic information; 3D view; checkCIF report

## Figures and Tables

**Fig. 1.**
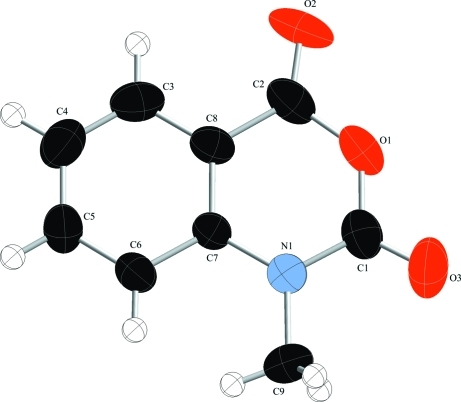
Thermal ellipsoid plot at 50% probability, the disordered hydrogen atoms were removed for clarity.

**Fig. 2.**
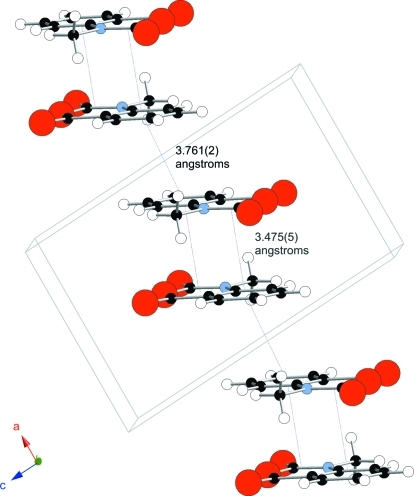
The packing diagram viewed along the b axis shows π-stacked dimers that are separated by 3.475 (5) Å (centroid to centroid shown as a blue dashed line). A staircase-like linear chain forms from additional π-stacking through the bezenoid rings (3.761 (2) Å). Oxygen atoms are shown in red, nitrogen atoms in blue, carbon atoms in red and the hydrogen atoms in white.

## supplementary crystallographic information

### Comment

1-Methyl-2*H*-3,1- benzoxazine-2,4(1*H*)-dione (*N*-methylisatoic anhydride, 1) is a key intermediate for the synthesis of a variety of compounds, such as agricultural chemicals, dyes/pigments flavors, fragrances, pharmaceuticals, ultraviolet absorbers, as well as esters, thioesters and amides of *N*-methylanthranilic acid (Kappe and Stadlbauer, 1981; Coppola, 1980; Shvekhgeimer, 2001). During the investigation of a novel *o*-aminoaryl oxazoline synthesis from the reaction of N-substituted isatoic anhydrides and 2-chloroethylamine hydrochloride in DMSO using an equimolar quantity of base, *o*-amino thiomethyl esters were observed as side products. In some cases, an *o*-amino thiomethyl ester was the major product (Hunt and Cherney, unpublished results).

The title compound is a planar molecule (Fig. 1). The bond distances are consistent with an aromatic system. The packing diagram, (Fig. 2) shows the aromatic rings form pi stacked dimers between the benzenoid ring and the 2,4 dicarbonyl oxazine ring, 3.475 (5) Å for both centroid to centroid distances (Crystalmaker ver. 2.1.2). The dimer forms a staircase-like linear chain through additional pi stacking between the benzenoid rings (3.761 (2) Å centroid to centroid).

### Experimental

During the course of the reaction of N-substituted isatoic anhydrides and 2-chloroethylamine hydrochloride, unreacted 1 was isolated from products via silica gel chromatography in a 98:2 dichloromethane/methanol mobile phase.  The 98:2 eluent was slowly evaporated to produce colorless rods and blocks of 1.  A block was chosen for X-ray analysis, the rods gave the same unit cell as the block.

### Refinement

The structure was solved using direct methods. The hydrogen atoms were positioned geometrically. A small improvement in the refinement occurred when the methyl hydrogens were modeled as 50% disordered due to free rotation about the C—N bond.

### Crystal data

**Table:**

C_9_H_7_NO_3_	*F*(000) = 368
*M**_r_* = 177.16	*D*_x_ = 1.495 Mg m^−^^3^
Monoclinic, *P*2_1_/*n*	Mo *K*α radiation, λ = 0.71073 Å
Hall symbol: -P 2yn	Cell parameters from 5577 reflections
*a* = 7.632 (2) Å	θ = 5.8–59.2°
*b* = 8.818 (2) Å	µ = 0.11 mm^−^^1^
*c* = 11.719 (3) Å	*T* = 296 K
β = 93.599 (4)°	Block, colorless
*V* = 787.1 (4) Å^3^	0.5 × 0.5 × 0.4 mm
*Z* = 4	

### Data collection

**Table:**

Bruker APEXII CCD diffractometer	2191 independent reflections
Radiation source: fine-focus sealed tube	1532 reflections with *I* > 2σ(*I*)
graphite	*R*_int_ = 0.029
ω and φ scans	θ_max_ = 30.1°, θ_min_ = 2.9°
Absorption correction: multi-scan (*SADABS*; Bruker, 2008)	*h* = −10→10
*T*_min_ = 0.902, *T*_max_ = 0.934	*k* = −9→12
13548 measured reflections	*l* = −16→16

### Refinement

**Table:**

Refinement on *F*^2^	Primary atom site location: structure-invariant direct methods
Least-squares matrix: full	Secondary atom site location: difference Fourier map
*R*[*F*^2^ > 2σ(*F*^2^)] = 0.052	Hydrogen site location: inferred from neighbouring sites
*wR*(*F*^2^) = 0.160	H-atom parameters constrained
*S* = 1.07	*w* = 1/[σ^2^(*F*_o_^2^) + (0.0717*P*)^2^ + 0.1822*P*] where *P* = (*F*_o_^2^ + 2*F*_c_^2^)/3
2191 reflections	(Δ/σ)_max_ < 0.001
118 parameters	Δρ_max_ = 0.20 e Å^−^^3^
0 restraints	Δρ_min_ = −0.21 e Å^−^^3^

### Special details

**Table:**

Geometry. All esds (except the esd in the dihedral angle between two l.s. planes) are estimated using the full covariance matrix. The cell esds are taken into account individually in the estimation of esds in distances, angles and torsion angles; correlations between esds in cell parameters are only used when they are defined by crystal symmetry. An approximate (isotropic) treatment of cell esds is used for estimating esds involving l.s. planes.
Refinement. Refinement of *F*^2^ against ALL reflections. The weighted *R*-factor wR and goodness of fit *S* are based on *F*^2^, conventional *R*-factors *R* are based on *F*, with *F* set to zero for negative *F*^2^. The threshold expression of *F*^2^ > σ(*F*^2^) is used only for calculating *R*-factors(gt) etc. and is not relevant to the choice of reflections for refinement. *R*-factors based on *F*^2^ are statistically about twice as large as those based on *F*, and *R*- factors based on ALL data will be even larger.

### Fractional atomic coordinates and isotropic or equivalent isotropic displacement parameters (Å^2^)

**Table:**

	*x*	*y*	*z*	*U*_iso_*/*U*_eq_	Occ. (<1)
O3	0.9373 (2)	0.59606 (17)	0.17004 (14)	0.0830 (5)	
O2	0.9649 (2)	1.0762 (2)	0.27147 (12)	0.0906 (6)	
O1	0.93997 (16)	0.83752 (18)	0.21847 (10)	0.0668 (4)	
N1	0.80524 (17)	0.76241 (15)	0.04367 (11)	0.0494 (3)	
C1	0.8953 (2)	0.7229 (2)	0.14315 (15)	0.0576 (4)	
C2	0.9119 (2)	0.9903 (2)	0.19846 (14)	0.0596 (5)	
C3	0.7855 (2)	1.1789 (2)	0.06087 (16)	0.0607 (5)	
H3	0.8214	1.2561	0.1112	0.073*	
C4	0.6984 (3)	1.2139 (2)	−0.04182 (18)	0.0662 (5)	
H4	0.6748	1.3144	−0.0613	0.079*	
C5	0.6463 (2)	1.0989 (2)	−0.11575 (15)	0.0591 (4)	
H5	0.5872	1.1228	−0.1853	0.071*	
C6	0.6794 (2)	0.95018 (19)	−0.08922 (13)	0.0488 (4)	
H6	0.6430	0.8742	−0.1404	0.059*	
C7	0.76789 (17)	0.91246 (16)	0.01464 (11)	0.0402 (3)	
C8	0.82028 (18)	1.02777 (18)	0.08998 (12)	0.0462 (4)	
C9	0.7552 (3)	0.6393 (2)	−0.0348 (2)	0.0780 (6)	
H9A	0.6927	0.6799	−0.1016	0.117*	0.50
H9B	0.6811	0.5691	0.0022	0.117*	0.50
H9C	0.8587	0.5878	−0.0567	0.117*	0.50
H9D	0.7956	0.5446	−0.0025	0.117*	0.50
H9E	0.8072	0.6554	−0.1063	0.117*	0.50
H9F	0.6297	0.6367	−0.0474	0.117*	0.50

### Atomic displacement parameters (Å^2^)

**Table:**

	*U*^11^	*U*^22^	*U*^33^	*U*^12^	*U*^13^	*U*^23^
O3	0.0874 (10)	0.0758 (10)	0.0840 (10)	0.0213 (8)	−0.0077 (8)	0.0243 (8)
O2	0.0825 (10)	0.1184 (14)	0.0673 (9)	−0.0039 (9)	−0.0231 (7)	−0.0386 (9)
O1	0.0639 (7)	0.0889 (10)	0.0455 (6)	0.0043 (7)	−0.0147 (5)	0.0028 (6)
N1	0.0527 (7)	0.0442 (7)	0.0499 (7)	0.0013 (5)	−0.0079 (5)	−0.0009 (5)
C1	0.0515 (9)	0.0646 (11)	0.0558 (9)	0.0072 (7)	−0.0025 (7)	0.0104 (8)
C2	0.0470 (8)	0.0819 (13)	0.0489 (8)	−0.0014 (8)	−0.0060 (6)	−0.0148 (8)
C3	0.0554 (9)	0.0509 (10)	0.0758 (11)	−0.0090 (7)	0.0049 (8)	−0.0169 (8)
C4	0.0670 (11)	0.0474 (10)	0.0839 (13)	−0.0007 (8)	0.0030 (9)	0.0084 (8)
C5	0.0597 (10)	0.0622 (11)	0.0549 (9)	0.0018 (8)	−0.0015 (7)	0.0128 (8)
C6	0.0505 (8)	0.0531 (9)	0.0418 (7)	−0.0028 (7)	−0.0048 (6)	−0.0009 (6)
C7	0.0368 (6)	0.0443 (8)	0.0393 (6)	−0.0016 (5)	0.0001 (5)	−0.0022 (5)
C8	0.0390 (7)	0.0530 (9)	0.0463 (7)	−0.0044 (6)	0.0003 (5)	−0.0095 (6)
C9	0.0984 (15)	0.0469 (10)	0.0854 (14)	0.0068 (10)	−0.0219 (11)	−0.0154 (9)

### Geometric parameters (Å, °)

**Table:**

O3—C1	1.201 (2)	C4—H4	0.9300
O2—C2	1.194 (2)	C5—C6	1.368 (2)
O1—C1	1.371 (2)	C5—H5	0.9300
O1—C2	1.382 (3)	C6—C7	1.3944 (19)
N1—C1	1.361 (2)	C6—H6	0.9300
N1—C7	1.3912 (19)	C7—C8	1.3888 (19)
N1—C9	1.459 (2)	C9—H9A	0.9600
C2—C8	1.450 (2)	C9—H9B	0.9600
C3—C4	1.373 (3)	C9—H9C	0.9600
C3—C8	1.397 (3)	C9—H9D	0.9600
C3—H3	0.9300	C9—H9E	0.9600
C4—C5	1.376 (3)	C9—H9F	0.9600
			
C1—O1—C2	125.43 (13)	C7—C8—C3	120.04 (14)
C1—N1—C7	122.51 (14)	C7—C8—C2	119.62 (15)
C1—N1—C9	116.62 (15)	C3—C8—C2	120.34 (15)
C7—N1—C9	120.81 (13)	N1—C9—H9A	109.5
O3—C1—N1	125.14 (18)	N1—C9—H9B	109.5
O3—C1—O1	117.80 (16)	H9A—C9—H9B	109.5
N1—C1—O1	117.06 (15)	N1—C9—H9C	109.5
O2—C2—O1	117.05 (18)	H9A—C9—H9C	109.5
O2—C2—C8	127.4 (2)	H9B—C9—H9C	109.5
O1—C2—C8	115.59 (14)	N1—C9—H9D	109.5
C4—C3—C8	120.15 (16)	H9A—C9—H9D	141.1
C4—C3—H3	119.9	H9B—C9—H9D	56.3
C8—C3—H3	119.9	H9C—C9—H9D	56.3
C3—C4—C5	119.44 (17)	N1—C9—H9E	109.5
C3—C4—H4	120.3	H9A—C9—H9E	56.3
C5—C4—H4	120.3	H9B—C9—H9E	141.1
C6—C5—C4	121.41 (16)	H9C—C9—H9E	56.3
C6—C5—H5	119.3	H9D—C9—H9E	109.5
C4—C5—H5	119.3	N1—C9—H9F	109.5
C5—C6—C7	119.98 (15)	H9A—C9—H9F	56.3
C5—C6—H6	120.0	H9B—C9—H9F	56.3
C7—C6—H6	120.0	H9C—C9—H9F	141.1
C8—C7—N1	119.63 (13)	H9D—C9—H9F	109.5
C8—C7—C6	118.98 (14)	H9E—C9—H9F	109.5
N1—C7—C6	121.39 (13)		
